# Endoscopic Ultrasound-Guided Gastroenterostomy for Malignant Gastric Outlet Obstruction: A Minimally Invasive Alternative to Palliative Surgical Bypass

**DOI:** 10.7759/cureus.59084

**Published:** 2024-04-26

**Authors:** Harsimran Kalsi, Terry L Jue

**Affiliations:** 1 Internal Medicine, UCF-HCA Florida North Florida Hospital, Gainesville, USA; 2 Gastroenterology, Mayo Clinic, Phoenix, USA

**Keywords:** palliative endoscopic procedure, minimally invasive endoscopic procedure, self-expanding metal stents, endoscopic ultrasound guided gastrojejunostomy, malignant gastric outlet obstruction

## Abstract

Gastric outlet obstruction is a mechanical obstruction to the flow of gastric contents to the intestines. The most common causes of malignant gastric outlet obstruction (MGOO) are pancreatic and gastric cancers. MGOO is associated with reduced quality of life and poor prognosis due to malnourishment from the inability to tolerate oral intake. Surgical gastrojejunostomy and endoscopic placement of enteral stents are palliative options with different advantages and disadvantages. We present a case of MGOO treated with endoscopic ultrasound-guided gastroenterostomy, a minimally invasive alternative to palliative surgical bypass.

## Introduction

Malignant gastric outlet obstruction (MGOO) is a mechanical obstruction to the flow of gastric contents into the intestines due to underlying malignancy either infiltrating or compressing the pylorus or duodenum [1]. The most common cause of MGOO worldwide is distal gastric cancer followed by pancreatic adenocarcinoma [1]. Patients usually present with chronic nausea, vomiting of undigested food, early satiety, and inability to eat orally leading to weight loss, dehydration, malnutrition, and poor quality of life [1,2]. The stomach has an inherent property of distending, consequently patients with MGOO present at an advanced stage after high-grade obstruction develops. MGOO is associated with markedly reduced quality of life and poor prognosis and may be palliated by either surgical or endoscopic interventions [1,2].

Surgical gastrojejunostomy (SGJ) involves bypassing the obstruction by forming an anastomosis between the stomach and the small intestine. SGJ may be performed laparoscopically [3]. SGJ provides durable luminal patency but requires postoperative recovery time, affecting oral intake and when chemotherapy may be resumed [4,5].

Endoscopic placement of self-expanding metal stents (SEMS) across the obstruction restores luminal patency to palliate MGOO [4-6]. SEMS has a high success rate, with early resumption of oral feed, shorter hospital stays, and low morbidity. In comparison to SGJ, endoscopically placed SEMS have a shorter period of luminal patency due to tumor infiltration causing stent occlusion or stent migration [4,6].

However, endoscopic placement of an SEMS may be preferred over SGJ for patients with poor performance status who are not good operative candidates and have short life expectancy [5].

Endoscopic ultrasound-guided gastroenterostomy (EUS-GE) has emerged as a novel technique for relieving obstruction across MGOO [7]. Like SGJ, EUS-GE bypasses MGOO by creating a tract between stomach and distal duodenum or proximal jejunum. A common approach to performing EUS-GE involves traversing the malignant stricture with a guidewire under fluoroscopic guidance and then placing a catheter over the wire to administer saline that distends the small bowel distal to the obstruction [8]. The EUS endoscope subsequently images the distended small bowel through the posterior wall of the stomach. Under ultrasound guidance, an enterostomy is created between the stomach and small intestine endoscopically, across which a 15 mm to 20 mm wide, 10 mm long, double-flanged lumen apposing metal stent (LAMS) is deployed [7,8]. Patients are able to resume oral intake the same day as the procedure and be potentially discharged [7,8].

We present a case of an 82-year-old woman with MGOO that was successfully relieved by EUS-GE. We will compare endoscopic SEMS to SGJ and propose that EUS-GE provides an additional, potentially superior option to benefit patients with MGOO.

## Case presentation

An 82-year-old woman was admitted with an inability to tolerate oral intake after four months of worsening early satiety, vomiting, and 9.07 kg weight loss.

The patient was afebrile, normotensive, with a normal heart rate, and on room air. The complete blood count, liver function tests, and creatinine were normal. At the time of admission, the patient weighed 49.4 kg with a BMI of 21.3 kg/m^2^.

**Figure 1:**
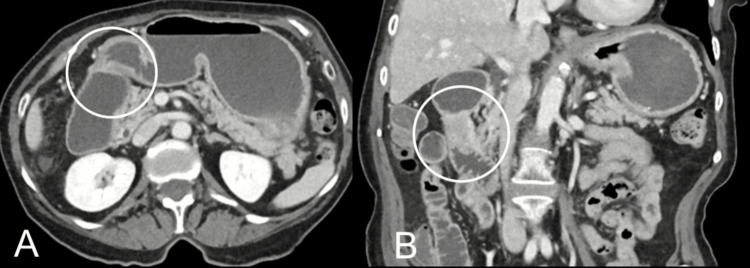
CT abdomen and pelvis with contrast (A) CT scan showing gastric outlet obstruction (transverse plane). (B) CT scan showing gastric outlet obstruction in the second part of the duodenum (coronal plane).

The patient underwent a CT scan of the abdomen and pelvis with contrast which suggested gastric outlet obstruction (GOO) due to focal mass-like thickening involving the second segment of the duodenum resulting in marked fluid distention of the gastric lumen and proximal duodenum (Figure 1). The patient underwent esophagogastroduodenoscopy (EGD) which showed a short stricture at the junction of the first and second portion of the duodenum, thus confirming GOO. EGD was combined with endoscopic ultrasound (EUS) imaging which ruled out any pancreatic masses. The biopsies collected from the duodenal mass confirmed the diagnosis of duodenal adenocarcinoma.

**Figure 2:**
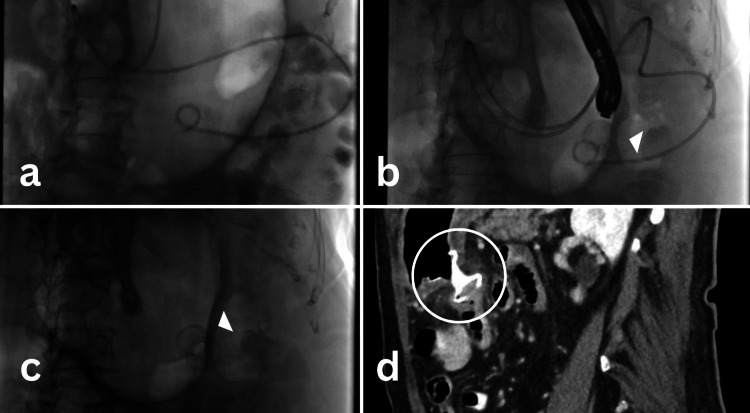
EUS-GE fluoroscopic deployment Images (a-c) and CT image post-procedure (d) (a) The picture shows the placement of the catheter across the stricture into the jejunum. This catheter is used to infuse 500-750 ml of normal saline to distend the small bowel distal to the stricture. (b) The picture shows the placement of LAMS using EUS scope. (c) The picture shows LAMS in place. (d). Post-procedural CT scan showing LAMS in place. EUS-GE: Endoscopic ultrasound-guided gastroenterostomy; LAMS: Lumen apposing metal stent

**Figure 3:**
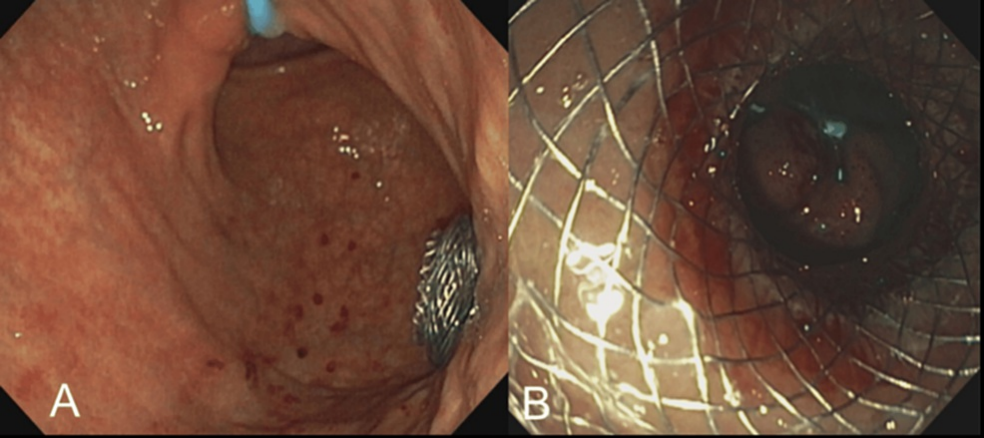
Post-EUS-GE endoscopic images (A) Post-procedural visualization of the LAMS in the stomach via an endoscope. (B) Visualization across the stent shows the small intestine. The blue irrigation catheter seen in the lumen of the small intestine confirms the successful creation of the gastroenterostomy. LAMS: Lumen apposing metal stent; EUS-GE: Endoscopic ultrasound-guided gastroenterostomy

The patient did not wish to undergo chemotherapy or surgical resection of the cancer. Surgical oncology was consulted and gastroenterology was re-consulted, who presented all the available options including SGJ, enteral stents, and EUS-GE. After extensive discussion, she elected to undergo EUS-GE rather than SGJ or endoscopic SEMS for palliation of MGOO as she did not wish to have recurrent hospitalizations. EUS-GE was successfully performed by placement of a 15 mm diameter fully covered 1 cm long LAMS (Hot Axios M00553550, Boston Scientific, Marlborough, USA) between the stomach and proximal jejunum (Figures 2, 3). The patient resumed a liquid diet on the day of the procedure and advanced to a soft diet before discharge. On follow-up, eight weeks later the patient was orally tolerating a solid food diet and regained 2.25 kg.

## Discussion

A systematic review of randomized controlled trials comparing SEMS to SGJ found similar rates of technical and clinical success (defined as resumption of oral intake), and no significant difference in rate or severity of complications [9]. Patients who underwent SEMS placement were able to resume oral intake and leave the hospital sooner than those who underwent SGJ, but there were higher re-intervention rates required in SEMS patients due to re-occlusion of the stent by the tumor. SGJ bypasses the malignant obstruction, which likely explains the lower rate of re-intervention compared to SEMS [5,9].

Ideally, EUS-GE provides the benefits of endoscopic intervention (earlier resumption of oral intake and a shorter hospital stay) with the benefits of SGJ (better patency with lower rates of re-intervention) [10]. A systematic review comparing EUS-GE to SEMS shows similar rates of technical success and length of hospital stay and significantly lower rates of stent occlusion and re-intervention (Table 1) [10].

**Table 1:** Comparing EUS-GE with enteral stents Credit: This table was created from the data presented by Boghossian et al. [10]. EUS-GE: Endoscopic ultrasound-guided gastroenterostomy; RD: Risk difference

	EUS-GE	Enteral stents	Risk difference
Technical success	93.33%	98.35%	RD=-0.01 (95% CI -0.06 to 0.04; p=0.68)
Clinical success	88.33%	78.02%	RD=-0.14 (95% CI -0.25 to -0.03; p=0.01)
Serious adverse events	11.66	31.32%	RD=-0.20 (95% CI -0.33 to 0.08; p=0.002)
Stent obstruction	3.33%	24.17%	RD=-0.20 (95% CI -0.29 to -0.12; p<0.01)
Stent obstruction due to tumor ingrowth	1.66%	16.48%	RD=-0.14 (95% CI -0.21 to -0.06; p<0.01)
Length of hospital stay	Mean difference=-1.42		(95% CI -0.31 to 3.14; p=0.11)
Symptom recurrence and reintervention	6.67%	28.57%	RD=-0.20 (95% CI -0.31 to -0.08; p<0.01)
30-day all-cause mortality	0.00%	6.92%	RD=-0.07 (95% CI -0.15 to 0.01; p=0.09)

A systematic review comparing SGJ to EUS-GE reported that SGJ had a significantly higher technical success rate (100% versus 91.4%). SGJ and EUS-GE had similar clinical success, adverse event rate, and reintervention rate [11]. EUS-GE did have a significantly shorter post-procedure hospitalization (five days) versus SGJ (Table 2) [11] and has been reported to allow earlier resumption of oral intake [12,13].

**Table 2:** Comparing EUS-GE with SGJ Credit: This table was created based on the data presented by Boghossian et al. [10]. EUS-GE: Endoscopic ultrasound-guided gastroenterostomy; SGJ: Surgical gastrojejunostomy; RD: Risk difference

	EUS-GE	SGJ	Risk difference
Technical success	91.41%	100%	RD -0.08 (95% CI -0.14 to -0.02; p<0.01)
Clinical success	86.71%	90.21%	RD -0.03 (95% CI -0.11 to 0.05; p=0.48)
Serious adverse events	11.72%	10.49%	RD -0.04 (95% CI -0.20 to 0.12; p=0.62)
Length of hospital stay	Mean difference: -5.11 days		(95% CI -8.51 to -1.71; p<0.01)
Symptom recurrence and reintervention	8.74%	10.53%	RD -0.04 (95% CI -0.13 to 0.06; p=0.44)
30-day all-cause mortality	4.69%	1.40%	RD of 0.00 (95% CI -0.03 to 0.04; p=0.86)

Reported causes for technical failure of EUS-GE are stent dislodgement during deployment, stent deployment into the peritoneum, and inability to distend a limb of the jejunum for the creation of an anastomosis [11,13].

A limitation of the data comparing EUS-GE to SEMS and SGJ is the absence of prospective, randomized studies comparing the three palliative options. The study designs should compare technical success, clinical success (resumption of oral intake), post-procedural time to resumption of oral intake, length of hospital stay, complications, severity of complications, re-intervention rates, and survival. Another limitation is that published outcomes are from high-volume tertiary centers with expertise in complex endoscopic and surgical procedures. Hence, the reproducibility of the reported outcomes may vary in clinical practice and palliative options should be guided by local expertise and patient preferences. While EUS-GE offers the potential combined advantages of endoscopic SEMS (earlier resumption of oral intake and a shorter hospital stay) with SGJ (better patency with lower rates of re-interventions), future studies should be conducted to provide data that guides the ideal palliative treatment of MGOO.

## Conclusions

EUS-GE is a novel minimally invasive technique for relieving obstruction across MGOO. EUS-GE bypasses MGOO by creating a tract between the stomach and distal duodenum or proximal jejunum. On comparing EUS-GE to SGJ, both have similar technical and clinical success. EUS-GE leads to shorter post-procedure hospital stays and early oral intake resumption when compared to SGJ. On comparing EUS-GE to enteral stents, EUS-GE has a lower rate of symptom recurrence, hospital admissions, and re-interventions.
